# Supplementary material for: Isolation of bacteria that catabolize abiotically synthesized tetroses via the formose reaction
Source: Sci Rep. 2025 Nov 29;16:734. doi: 10.1038/s41598-025-30247-3 (PMC12780249; doi:10.1038/s41598-025-30247-3)

Isolation of bacteria that catabolize abiotically synthesized tetroses via the formose reaction

Kosei Kawasaki[1], Kensuke Igarashi[1], Hiro Tabata[2,3], Hiroaki Nishijima[2], Shuji Nakanishi[2,4], and Souichiro Kato[1,2,5]*

[1] Bioproduction Research Institute, National Institute of Advanced Industrial Science and Technology, 2-17-2-1, Tsukisamu-Higashi, Toyohira, Sapporo, 062-8517 Japan; [2] Research Center for Solar Energy Chemistry, Graduate School of Engineering Science, Osaka University, 1-3 Machikaneyama, Toyonaka, Osaka, 560-8531 Japan; [3] Presidential Endowed Chair for Platinum Society, The University of Tokyo, 7-3-1 Hongo, Bunkyo-Ku, Tokyo, 113-8656 Japan; [4] Innovative Catalysis Science Division, Institute for Open and Transdisciplinary Research Initiatives (ICS-OTRI), Osaka University, Suita, Osaka 565-0871, Japan; [5] Department of Biotechnology, Graduate School of Agricultural and Life Sciences, The University of Tokyo, 1-1-1 Yayoi, Bunkyo-ku, Tokyo, Japan.

Corresponding author

*Souichiro Kato; E-mail: sou-kato@g.ecc.u-tokyo.ac.jp

Supplementary Fig. S1. Concentrations of C4 sugars (A) and C1-C3 compounds (B) under the uninoculated control. The minimal medium supplemented with 5% of the abiotically synthesized sugars were incubated at 30°C with agitation (180 rpm). Data are presented as the means of three independent cultures. Error bars represent standard deviations.

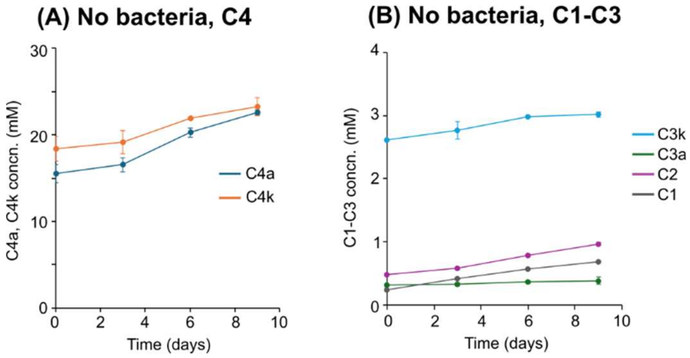

Supplementary Fig. S2. Consumption of C1-3 compounds in the abiotically synthesized sugars by the tetrose-utilizing isolates. Each isolate was cultured in the minimal medium supplemented with 5% of the abiotically synthesized sugars and the changes in the concentrations of each sugar species were determined. The data were obtained from the same culture experiments depicted in Fig. 3. Data are presented as the means of three independent cultures. Error bars represent standard deviations.

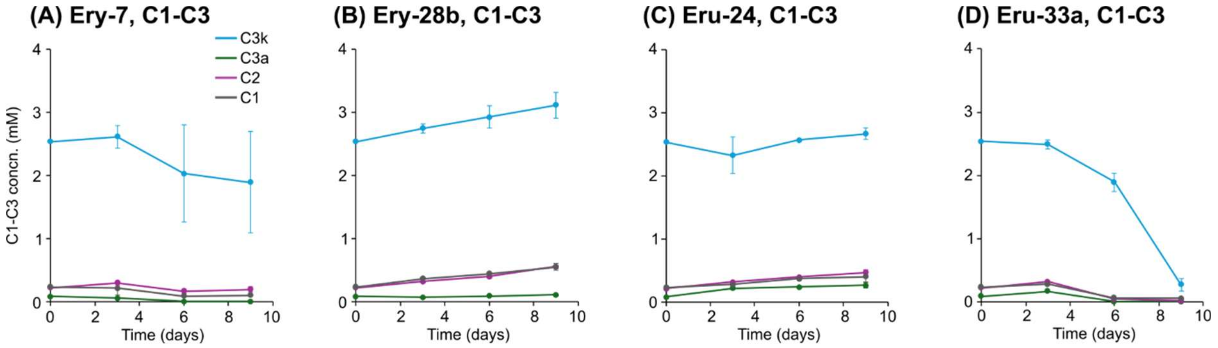

Supplementary Fig. S3. Catabolic pathways for C4 sugar alcohols identified in different bacterial strains to date. (A) An erythritol catabolic pathway identified in *Brucella abortus* (Geddes *et al*., 2103; Barbier *et al.*, 2014). (B-D) Catabolic pathways for D-threitol, erythritol, and L-threitol, respectively, identified in *Mycolicibacterium smegmatis* (Huang *et al.*, 2015).

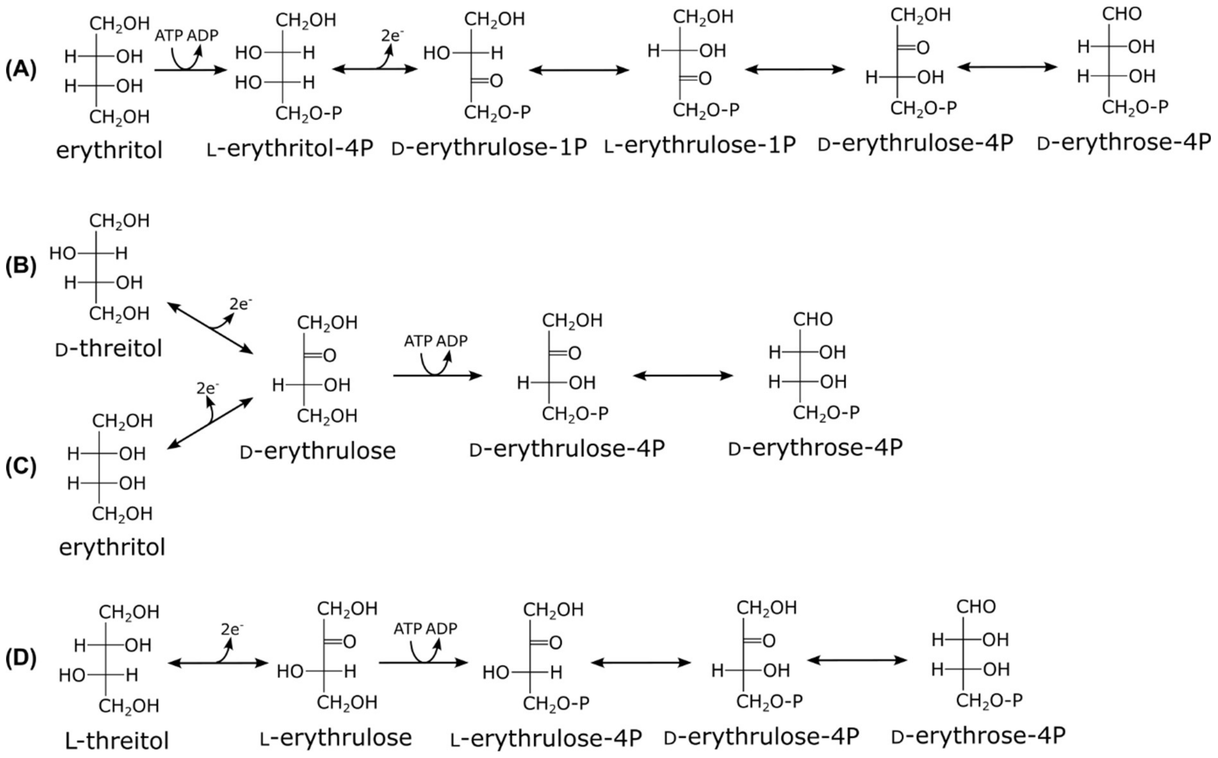

Supplement: Supplementary file 1 — Supplementary Material 1 [file 41598_2025_30247_MOESM1_ESM.pdf]
